# Supplementary material for: Characterization of Phosphorus Species in Human Dentin by Solid-State NMR
Source: Molecules. 2020 Jan 3;25(1):196. doi: 10.3390/molecules25010196 (PMC6983145; doi:10.3390/molecules25010196)
Supplement: Supplementary file 1 [file molecules-25-00196-s001.pdf]

Supplementary Information for

Characterization of Phosphorus Species in Human Dentin by Solid-State NMR

Yi-Ling Tsai [1], Meng-Wei Kao [1], Shing-Jong Huang [2], Yuan-Ling Lee [3,*], Chun-Pin Lin [3,*] and Jerry Chun Chung Chan [1,*]

[1] Department of Chemistry, National Taiwan University, No. 1, Sec. 4, Roosevelt Road, Taipei, Taiwan
[2] Instrumentation Center, National Taiwan University, No. 1, Sec. 4, Roosevelt Road, Taipei, Taiwan
[3] School of Dentistry, National Taiwan University Hospital, National Taiwan University, No. 7, Chung San South Road, Taipei, Taiwan

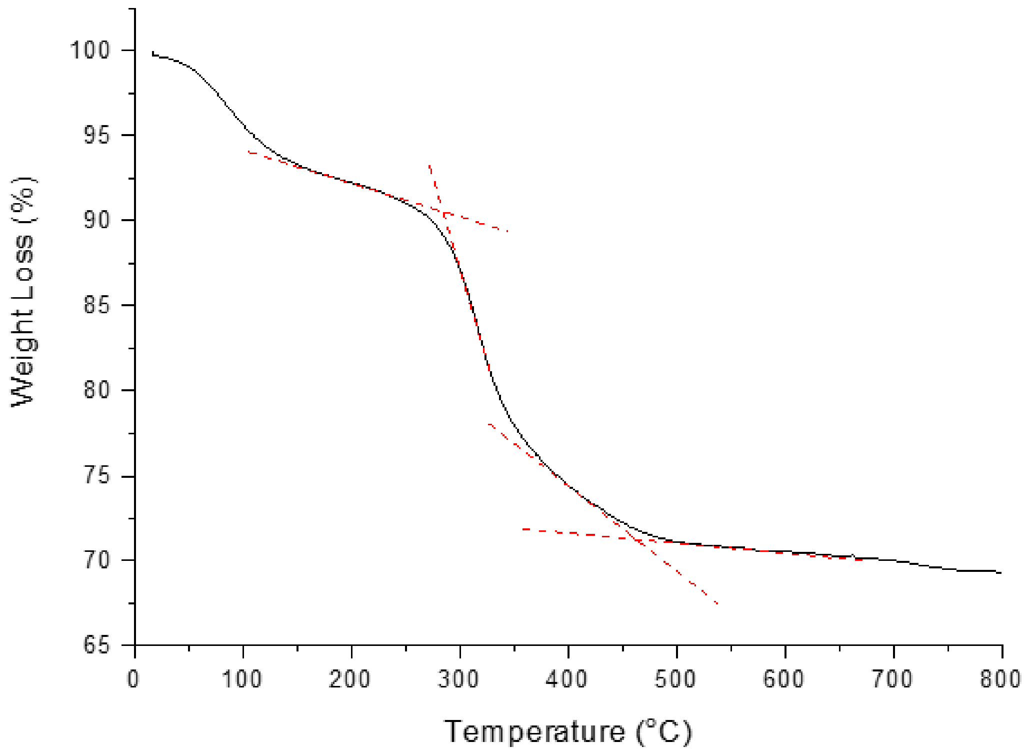

Fig. S1. Typical TGA profile obtained for human dentin samples. The weight loss attributed to the removal of organic matter was estimated by the vertical difference of the two cross points.

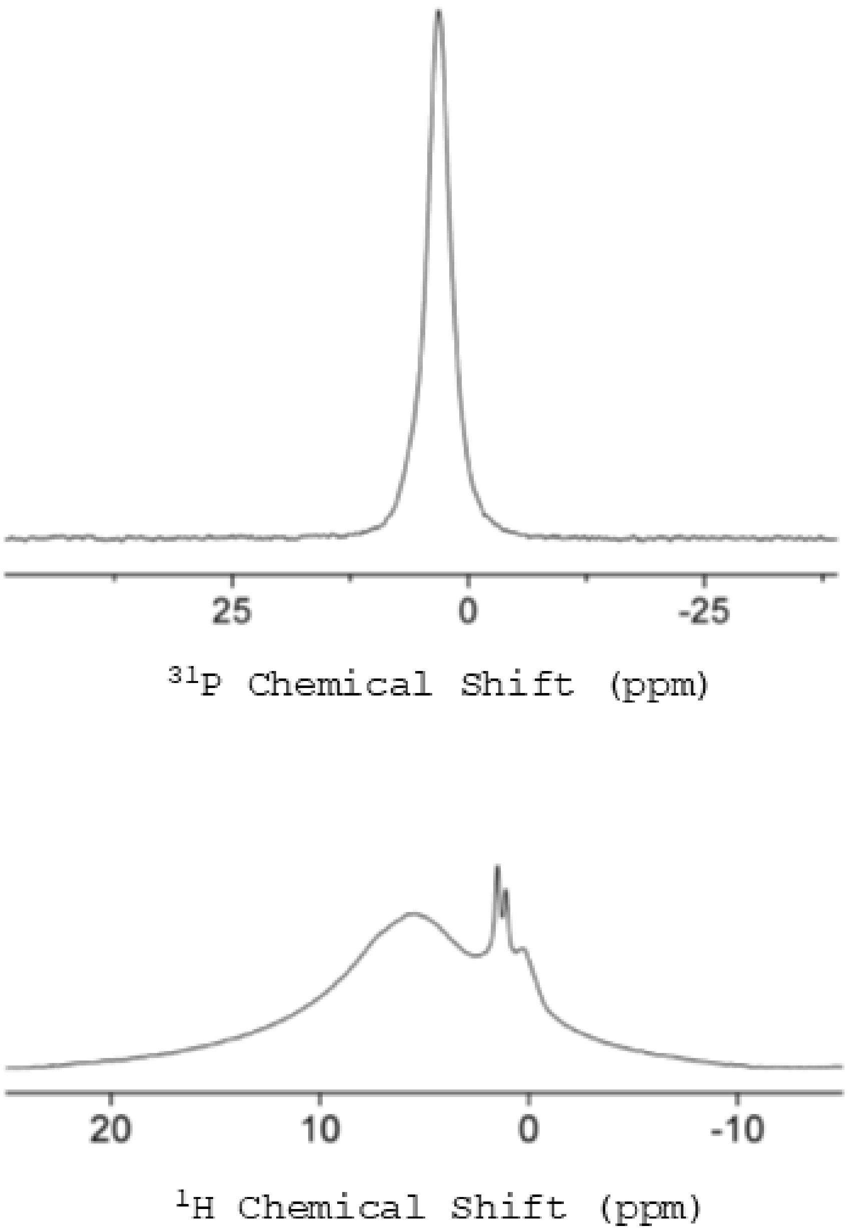

Fig. S2. Typical solid-state NMR spectra acquired under magic-angle conditions (MAS) for human dentin samples at a spinning frequency of 10 kHz.

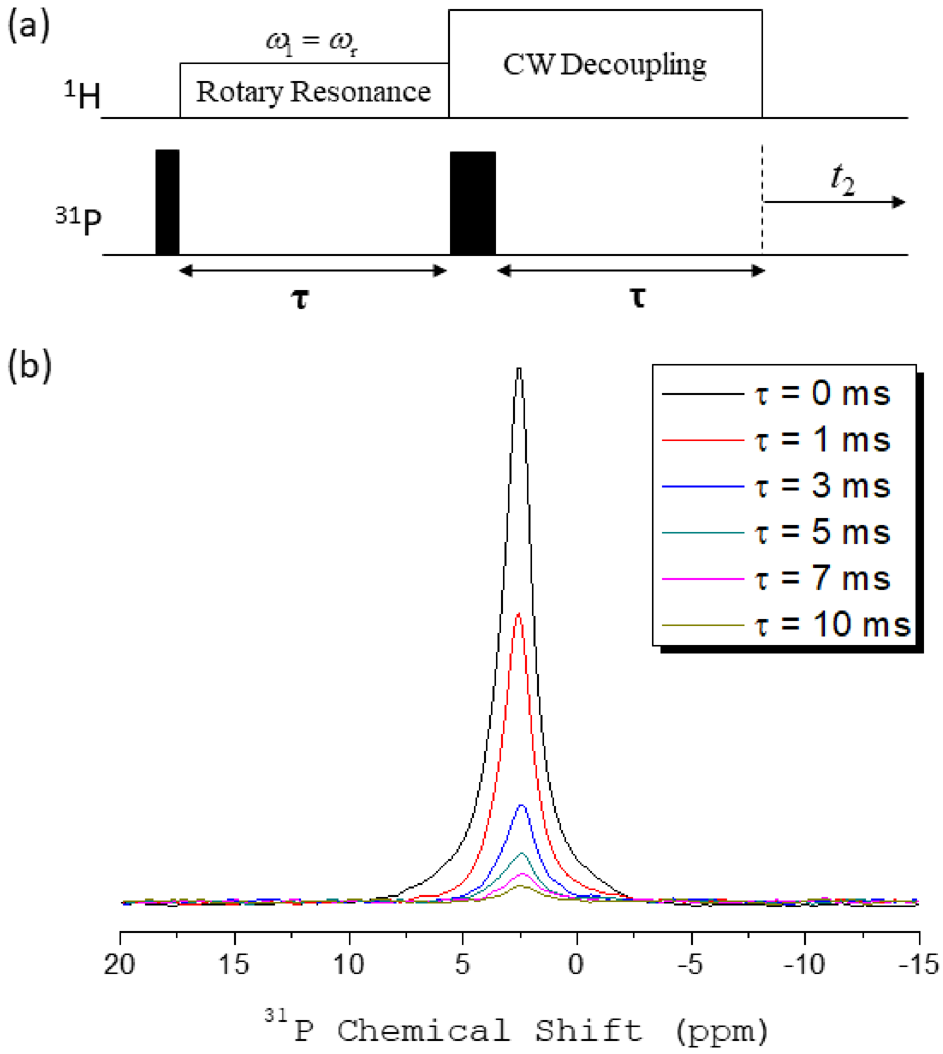

Fig. S3. (a) The pulse sequence of the dipolar dephasing experiment based on the rotary resonance technique. The ^{31}P $\pi/2$ and π pulses were set to 5 and 10 μs long, respectively. ^{1}H decoupling at a field strength of 80 kHz was used. The recycle delay was set to 600 s. **(b)** The spectra acquired for HAp indicated that the signal of the orthophosphate group in close proximity of the hydroxyl group could be suppressed effectively when the dephasing period is $\geq$ 10 ms.

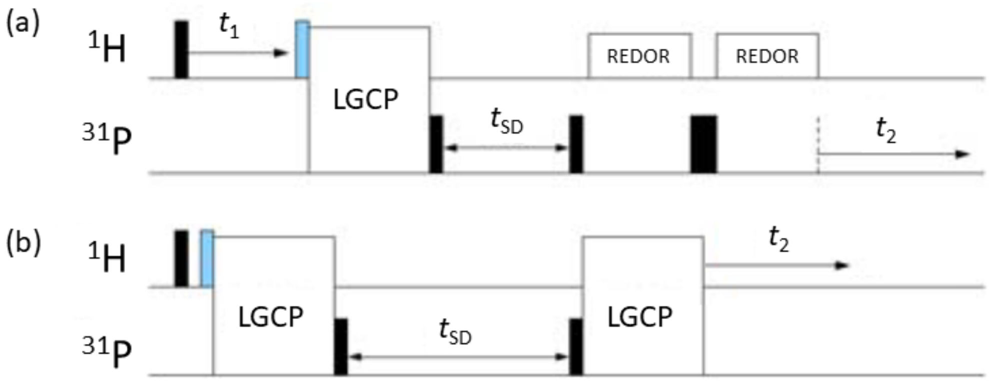

Fig. S4. Pulse sequences employed for the ^{31}P spin diffusion experiments. The spinning frequency was set to 10 kHz. (**a**) the 2-D technique used to probe the spin diffusion between the phases of HAp and HDAp, and that between dentin-ACP and HDAp; (**b**) the 1-D technique used to probe the spin diffusion between HAp and dentin-ACP. For the Lee-Goldburg CP (LGCP) period, the ^{1}H nutation frequency and the resonance offset were set equal to 50 and 35.35 kHz, respectively, to fulfill the LG irradiation condition and the ^{31}P nutation frequency was ramped through the Hartmann-Hahn matching condition. The flip angle of the pulse right before the LGCP block was adjusted to 144.7 deg so that the spin-temperature inversion can be realized by phase alternating the first ^{1}H $\pi/2$ pulse. The contact time of the LGCP period was set to 2.5 ms. The recycle delay was set to 2 s. For the REDOR block, the π pulse was set to 10 μs. The total REDOR dephasing time was set to 2 ms.

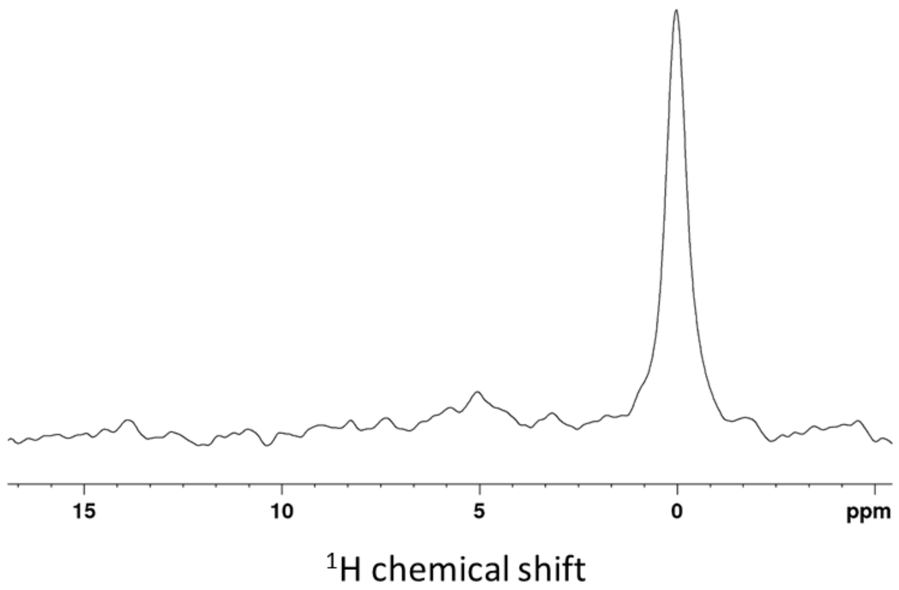

Fig. S5. Typical ^{1}H spectrum acquired with the pulse sequence shown in **Fig. S4(b)**. The first LGCP contact time was set to 12 ms to enhance the initial HAp signal. The contact time for the second CP block was set to 2 ms. The t_{SD} was varied to probe the transfer between the HAp and dentin-ACP signals. The data are shown in **Figure 6(a)** of the main text.

(a)

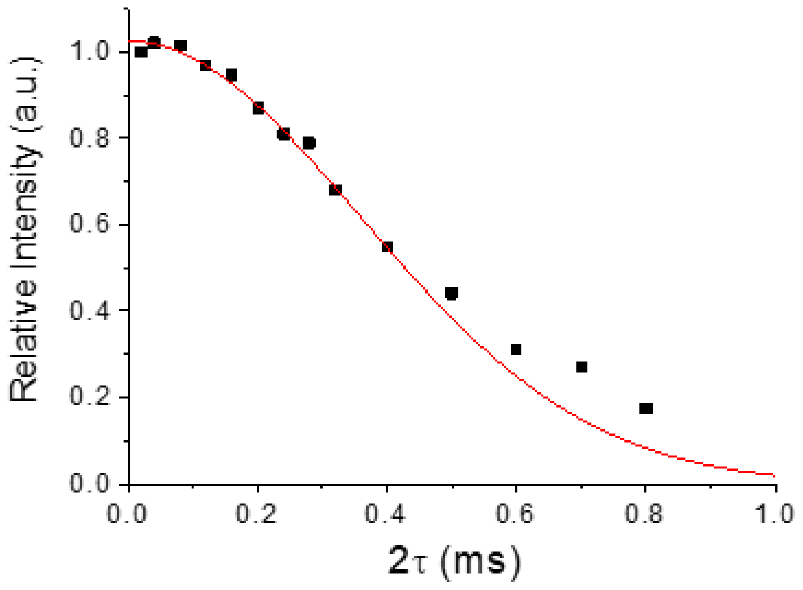

(b)

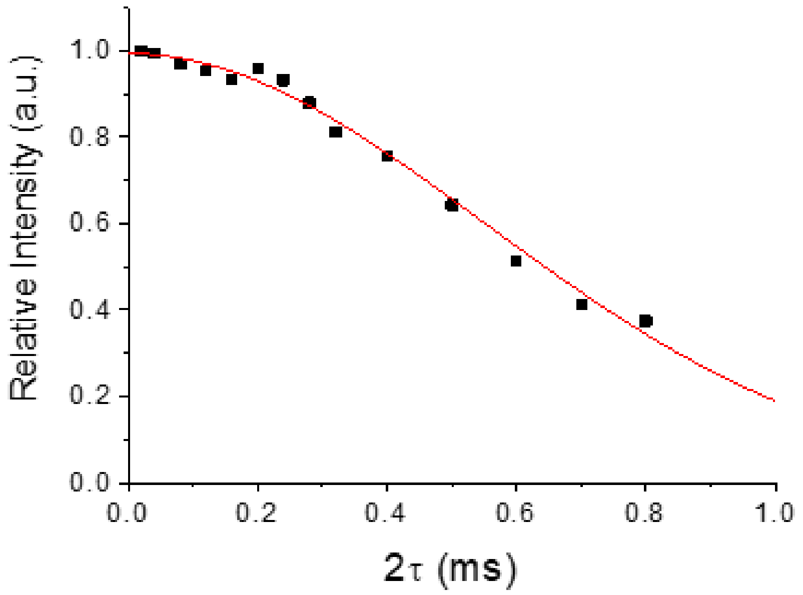

Fig. S6. Signal modulation of the spin-echo experiment as a function of the inter-pulse delay under static conditions. (**a**) HAp; (**b**) ACP. The data were fitted by a Gaussian decay, from which the M_2 values were extracted.

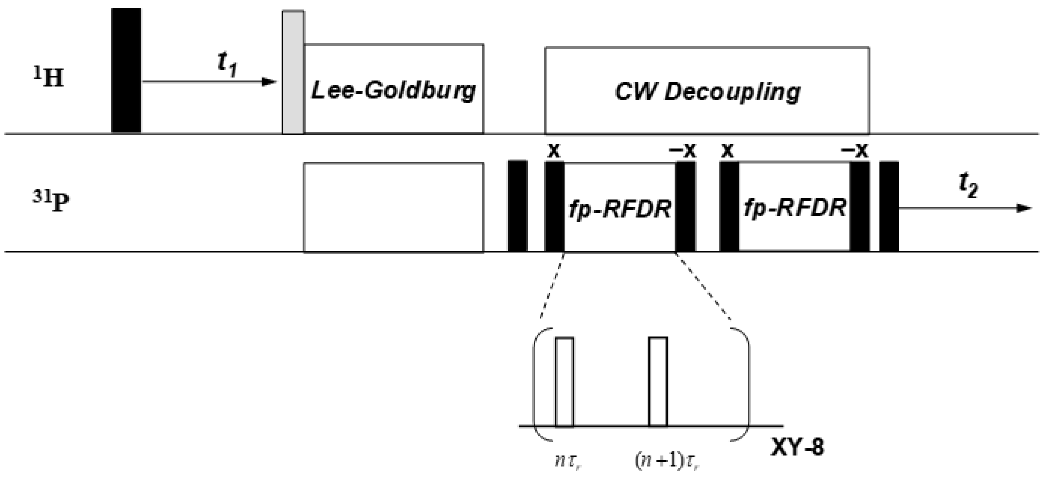

Fig. S7. Pulse sequence for the DQ-filtered HETCOR experiments. The flip angle of the pulse Lee-Goldburg condition during the CP contact time was set as described in the caption of Fig. S4. For the fp-RFDR period, the ^{31}P π pulses were set to 30 μs. The π pulse trains were phase cycled according to the XY-8 scheme. The ^{31}P $\pi/2$ pulses were set to 5 μs. The DQ reconversion period (the first fp-RFDR block flanked by two $\pi/2$ pulses) was set equal to the DQ excitation period. The ^{1}H decoupling was set to 85 kHz during the DQ excitation/reconversion periods.

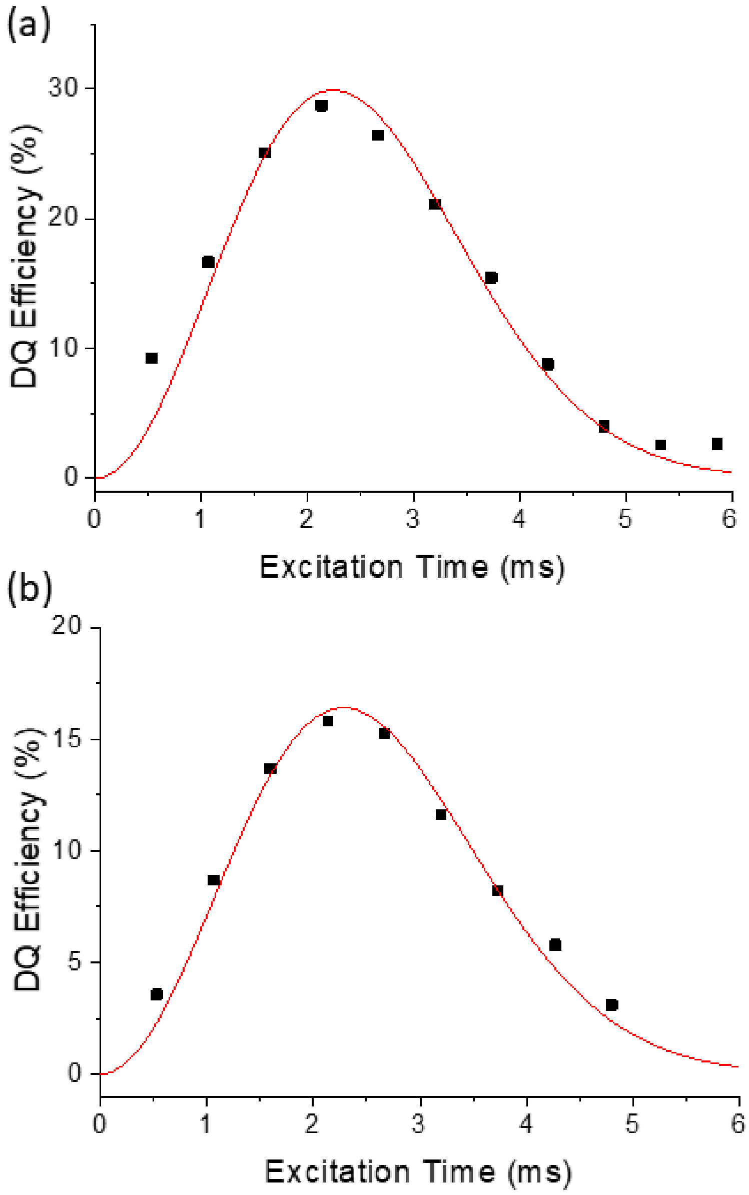

Fig. S8. Signal modulation of the DQ filtered HETCOR experiments as a function of the excitation time. (**a**) HAp; (**b**) ACP. The data were fitted by Eq. (3) of the main text.

Table S1. Summary of the NMR parameters extracted from the $^{31}P\{^1H\}$ variable contact-time HETCOR measurements of the human dentin samples.

Sample		M_0	τ_{CP} (ms)	$T_{1\rho}$ (ms)
R_{17}	apatite	1.00	4.6 ± 0.2	--
	dentin-ACP	0.85	0.48 ± 0.04	26 ± 9
R_{19}	apatite	1.00	5.0 ± 0.8	--
	dentin-ACP	0.81	0.58 ± 0.09	17 ± 4
R_{21}	apatite	1.00	3.6 ± 0.2	--
	dentin-ACP	0.62	0.52	10
R_{22}	apatite	1.00	5.3 ± 0.3	--
	dentin-ACP	0.81	0.56	20
R_{31}	apatite	1.00	4.8 ± 0.2	--
	dentin-ACP	0.84	0.56	18
R_{51}	apatite	1.00	4.3 ± 0.2	--
	dentin-ACP	0.70	0.59 ± 0.3	16 ± 2
